# Challenges to Optimize Charge Trapping Non-Volatile Flash Memory Cells: A Case Study of HfO_2_/Al_2_O_3_ Nanolaminated Stacks

**DOI:** 10.3390/nano13172456

**Published:** 2023-08-30

**Authors:** Dencho Spassov, Albena Paskaleva

**Affiliations:** Institute of Solid-State Physics, Bulgarian Academy of Sciences, Tzarigradsko Chaussee 72, 1784 Sofia, Bulgaria; d.spassov@issp.bas.bg

**Keywords:** charge trapping flash memory, HfO_2_/Al_2_O_3_ stacks, non-volatile memory, atomic layer deposition (ALD), high-k dielectrics

## Abstract

The requirements for ever-increasing volumes of data storage have urged intensive studies to find feasible means to satisfy them. In the long run, new device concepts and technologies that overcome the limitations of traditional CMOS-based memory cells will be needed and adopted. In the meantime, there are still innovations within the current CMOS technology, which could be implemented to improve the data storage ability of memory cells—e.g., replacement of the current dominant floating gate non-volatile memory (NVM) by a charge trapping memory. The latter offers better operation characteristics, e.g., improved retention and endurance, lower power consumption, higher program/erase (P/E) speed and allows vertical stacking. This work provides an overview of our systematic studies of charge-trapping memory cells with a HfO_2_/Al_2_O_3_-based charge-trapping layer prepared by atomic layer deposition (ALD). The possibility to tailor density, energy, and spatial distributions of charge storage traps by the introduction of Al in HfO_2_ is demonstrated. The impact of the charge trapping layer composition, annealing process, material and thickness of tunneling oxide on the memory windows, and retention and endurance characteristics of the structures are considered. Challenges to optimizing the composition and technology of charge-trapping memory cells toward meeting the requirements for high density of trapped charge and reliable storage with a negligible loss of charges in the CTF memory cell are discussed. We also outline the perspectives and opportunities for further research and innovations enabled by charge-trapping HfO_2_/Al_2_O_3_-based stacks.

## 1. Introduction

**Figure 1:**
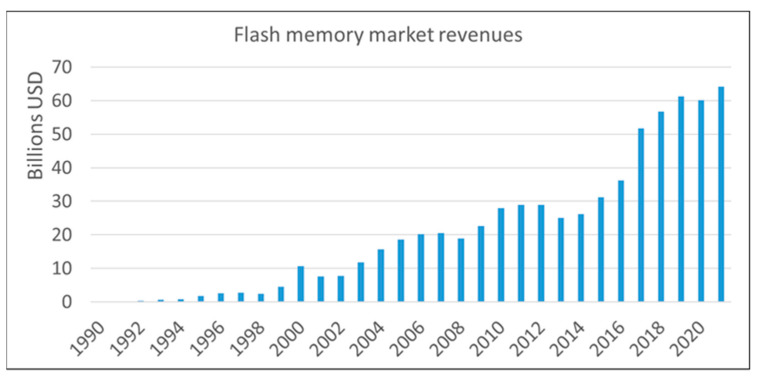
Flash memory market trends. Data from 1990 to 2006 by [1], from 2007 to 2012 from [2] and from 2013 to 2021 by [3].

Flash memory is an electronic, non-volatile information storage device that can be electrically erased and reprogrammed. Ideally, the information stored in such a device should be preserved for long when the power is switched off. Flash memories are the primary means to realize low-cost and high-density data storage needed for all major end-user gadgets (smartphones, PCs, USBs, medical devices, electronic games, etc.). The ever-widening field of possible applications made flash memories the fastest-growing product in the history of the semiconductor market (Figure 1).

**Figure 2:**
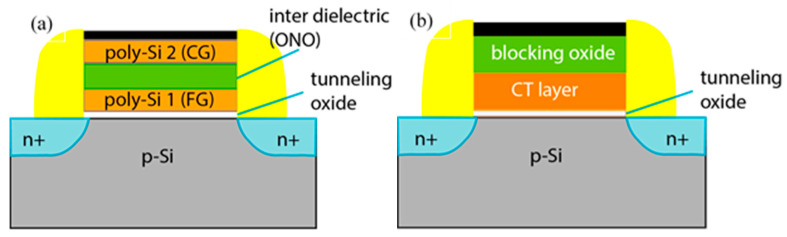
Schematic comparison between basic MOS transistor structures in floating gate (**a**) and charge trapping memory devices (**b**).

For a long time, the dominant flash NVM technology was the floating gate (FG) memory cell [4,5]. The idea for the FG memory cell was proposed by Kahng and S.M. Sze in 1967 [6]. Its operation principle is based on charge storage in an electrically isolated floating poly-Si gate. This floating gate is stacked in between a thin tunnelling (6–7 nm) oxide and interpoly dielectric (10–13 nm) (Figure 2a). By applying a pulse to the control gate, the electrons are injected from the transistor’s channel to the floating gate and stored there until the pulse with opposite polarity forces them out of the floating gate. The presence/absence of electrons on the floating gate changes the threshold voltage, thus forming the memory window. The charges stored in the electrically isolated floating gate remain there for a long time, thus defining the non-volatile character of memory. The increasing demands for larger volumes of stored data cause an aggressive down-scaling of FG cell sizes. As a consequence, some of the intrinsic limitations of floating gate technology have been reached, e.g.,: (i) the thickness scaling of tunnel oxide and inter-poly dielectric layer compromises the reliability; (ii) a significant decrease in the number of electrons accumulated in FG as its dimensions decrease; (iii) it is difficult to maintain a high coefficient of capacitive coupling of the control gate to the floating gate; (iv) the parasitic capacitance between adjacent cells leading to data interference becomes important, etc.

Therefore, several new approaches to achieve a non-volatile programmable memory effect have been proposed: ferroelectric field effect transistor, resistive switching memory, nanoelectromechanical memory, spin-transfer torque memory, phase change memory, etc. All these new memory concepts are classified as emerging memories and rely on distinctly different physical phenomena and principles than currently used. For most of them, the architecture of the device/memory cell, as well as the materials, are quite different than those already adopted in the microelectronic industry, and there are a lot of problems with making them compatible with the current technology, which eventually requires abandoning the CMOS paradigm. A comprehensive review of the operation principles, advantages, and shortcomings of these new NVM concepts can be found in [7]. Still, none of the above-mentioned innovative devices and technologies has been identified as the most prospective and clear winner to replace CMOS–based memory cells.

In summary, new computing and data storage paradigms (like neuromorphic or quantum), novel architectures and devices using charges or, in the longer term, alternative state variables (e.g., spin, magnon, phonon, photon, etc.) are required to scale information processing technology substantially beyond that attainable by the ultimately scaled CMOS [8]. While developing these new technological approaches and paving the way for their adoption by the industry, there are still innovations within the current technology paradigm, which have been implemented to increase the bit density of NVM. These are, for example, an increase in the number of up to 4 bits per memory cell and replacing the floating gate cell with a charge trap cell [9].

Charge trapping flash (CTF) NVMs (Figure 2b) are a promising alternative to the conventional floating gate technology because they involve a more simplified process flow accompanied by better operation characteristics, e.g., improved retention and endurance, lower power consumption, higher program/erase (P/E) speed [10,11,12]. The charge-trapping memory is proposed by H. A. R. Wegner et al. [13]. CTF operation is similar to the floating gate cell, but the charge in CTF is stored in spatially discrete traps in the band-gap of the dielectric layer instead of the conducting floating poly-Si gate. This mode of charge storage offers a significant advantage over FG because the discharge of the whole stored charge is prevented in the case of an isolated defect in the tunnel oxide (hence the leakage path), and only charges stored in traps adjacent to the leakage path may be lost. The architecture of CT memory is very similar to MOSFET. Hence, it is compatible with CMOS technology. The importance of CTF became undeniable when flash memories switched from scaling horizontally to stacking vertically. Flash products have already overcome the 2D limitations by aggressively implementing 3D memory cell structures—72–96 layers of NAND memory cells have already been demonstrated [8]. The use of CT-NVM is also favourable in Vertical-NAND flash memory technology as it is more easily stacked vertically [14].

The most important part of the CT memory is the charge-trapping stack (Figure 2b). It consists of three layers—a charge trapping layer (CTL), where the charges are stored in traps. CTL is stacked in between the tunnelling and blocking oxides. Tunnelling oxide (TO) is used for more efficient injection of charges from the substrate/FET channel to CTL to prevent the trapped charges from back tunnelling to the substrate and to improve the retention characteristics. On the other hand, to form a potential barrier against the undesirable movement of electrical charges (holes/electrons) to and from the gate electrode, a thick enough blocking oxide (BO) should be introduced, and its band offset with the CTL should be of sufficient height. Therefore, the use of SiO_2_ with its band-gap Eg of about 9.1 eV both as a blocking and a tunnelling oxide is a natural choice.

Moreover, recent technology allows SiO_2_ to be grown with very low densities of defects and traps, which may participate in the process of charge loss. In the current CTFs, Si_3_N_4_ is used as a CTL because Si_3_N_4_ provides a sufficiently high density of trapping sites. This charge trapping stack consisting of SiO_2_ (TO)-Si_3_N_4_ (CTL)-SiO_2_ (BO) tri-layer is usually referred to as an ONO stack. The ONO suffers from the trade-off between programming speed and retention. On the one hand, to enhance program/erase (P/E) speed, a thinner TO is required. On the other hand, thicker TO ensures better retention. The implementation of high-k materials in CTF is expected to overcome some of the problems arising from the down-scaling of CTF and extend the applicability of this technology [15].

In this review, we summarize and give a more general view of our systematic studies of metal/blocking oxide (BO)/high-k charge trapping layer (CTL)/tunnel oxide (TO)/Si (MOHOS) structures with HfO_2_/Al_2_O_3_-based CTL prepared by ALD considered for application in CTF memories. The work is organized as follows. Section 2 results on the density and energy location of traps, charge trapping and storage characteristics, leakage currents in HfO_2_/Al_2_O_3_-based CTLs, and their dependence on the composition of CTL and annealing in O_2_ are summarized. Section 3 considers the introduction of tunnelling and blocking oxide in the stack and their influence on the electrical behaviour of memory cells. Finally, in Section 4, the main conclusions of the study, as well as perspectives for further improvement and future applications of HfO_2_/Al_2_O_3_-based charge trapping stacks, are outlined.

## 2. Charge Trapping Layer

As mentioned above, high-k dielectric materials have been considered to replace Si_3_N_4_ in conventional CTFs [15,16]. HfO_2_-based high-k dielectric layers attracted much attention in the last two decades due to their importance as gate dielectrics. Respectively, the intensive studies of their properties and technology approaches to improve them resulted in their adoption by CMOS technology and successful application in Intel Penryn and Samsung A7 processors. Generally, HfO_2_ is a trap-rich material—a property that is undesired for high-performance logic applications (such as CPUs). Therefore, dedicated measures should be undertaken to reduce the density of electrically active defects to meet performance requirements. However, the high density of traps next to the relatively high dielectric constant, large conduction band offsets with Si and tunnel oxide, as CMOS compatibility made HfO_2_-based dielectrics a very attractive alternative to supersede the conventional Si_3_N_4_ as CTL in CTFs. The higher dielectric constant ensures robust data storage because it enables the storage of more electrons without increasing the applied field. It was shown that the 2 nm HfO_2_ layer has a better charge trapping efficiency than 7 nm Si_3_N_4_ [17]. Another advantage of using high-k dielectrics as CTL is that there is a large room to modify and tailor their properties toward meeting the specific requirements of the given application. For example, the charge storage characteristics could be significantly boosted by proper treatments, e.g., annealing steps, UV irradiation, etc. [18,19,20]. Doping/mixing with other elements was a very efficient way to modify the density and spatial and energy location of electrically active defects. Bandgap engineering of the CTL by introducing Al in HfO_2_ or stacking HfO_2_ with Al_2_O_3_ has been suggested [21,22,23,24], resulting in an enhancement in memory performance and reliability.

Moreover, the introduction of Al in high-k dielectrics is known to increase crystallization temperature [25]. CTL needs to be amorphous as the grain boundaries in crystalline layers may result in increased leakage currents. The increase in the number of the HfO_2_/Al_2_O_3_ interfaces has been reported to improve the charge-trapping ability of devices assigned to interdiffusion at the HfO_2_/Al_2_O_3_ interface and the creation of additional defects [26]. However, it appeared that the thickness of the layers and the number of interfaces should be carefully optimized as the performance of the cell could deteriorate by the electrostatic repulsion between the trapped charges [27]. The possibility to combine the HfO_2_/Al_2_O_3_ dielectric stacks with high-mobility channel materials (e.g., SiGe [28], GaAs [29,30], InP [31], In_2_Ga_2_ZnO_7_ [32]) as well as with 2D materials, (e.g., black phosphorous [33] and MoS_2_ [24,34]) opens up new horizons for their implementation in emerging applications such as thin film transistors, non-volatile memory devices for flexible and transparent electronics, etc.

For efficient and reliable trapping, a high density of traps is important, but these traps should be deep enough. Therefore, to get control and optimize the operation of CTF memory cells, a thorough understanding of the origin of traps and their spatial and energy location in the charge trapping layer should be acquired. The deep insight into the trapping kinetics and storage paves the way to successful process optimization and robust memory cell performance. In addition, the leakage current has to be low—a requirement that is quite challenging to satisfy simultaneously with the high density of intrinsic traps. Therefore, our efforts have aimed to produce charge-trapping layers with a high density of deep traps while preserving a low leakage current.

### 2.1. Density and Energy Location of Traps

**Figure 3:**
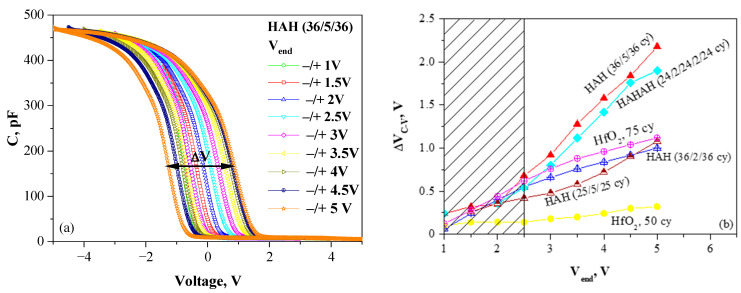
(**a**) C-V curves of HAH (36/5/36) sample measured in different voltage ranges (−V_end_ → +V_end_ → −V_end_). The memory window ΔV_C-V_ is shown. (**b**) Dependence of memory window ΔV_C-V_ on V_end_ for different sample structures (Reprinted with permission from [18]. Copyright 2015 American Chemical Society). (H denotes the HfO_2_ sublayer, and A denotes the Al_2_O_3_ one. For each sublayer, the number of ALD cycles is given).

As a first step in the optimization process, the impact of Al introduction in HfO_2_ on traps’ density and energy location has been investigated [18]. Several samples with different thicknesses and Al content have been deposited by ALD and compared to pure HfO_2_. The charge-trapping and the ability of a stack to store a charge is evaluated by measuring the C-V hysteresis ΔV_C-V_ in dependence on the end voltage V_end_ of the measured C-V curves (Figure 3a). ΔV_C-V_ is usually called a memory window. The detailed investigations and analysis of the obtained results [18] reveal two kinds of trapping processes: (i) The first process is irreversible trapping due to traps generated by the high electric field stress. This process is undesired because it results in permanent damage and progressive structure degradation. It has been established that it is independent of doping, which allows the conclusion that it occurs in HfO_2_-related defects; (ii) The second process is reversible trapping, i.e., the charges could be reversibly captured in and erased from the traps under proper biasing conditions. This is the reversible program/erase cycle, which defines the main principle of operation of CTFs.

The results have revealed that the C-V hysteresis, hence the density of trapped charge, depends very strongly on the layer thickness and a doping level (Figure 3b), and the findings could be summarized as follows: (i) The trapping is stronger in the thicker samples; (ii) lightly doped (2 cy Al_2_O_3_) layers have lower trap density compared to pure HfO_2_; and, more highly doped (4 or 5 cy Al_2_O_3_) layers reveal higher trap density, which is one of the condition for the efficient charge trapping and storage. The density of traps for more highly doped layers has been estimated in the range of N_t_ = (4–5) × 10^19^ cm^−3^; which is higher than the reported density of deep traps in Si_3_N_4_ of about 10^19^ cm^−3^ and required for a robust operation of CTF cell [35]. The centroid of the trapped charge is estimated at 4.95 nm, which coincides with the location of Al doping.

**Figure 4:**
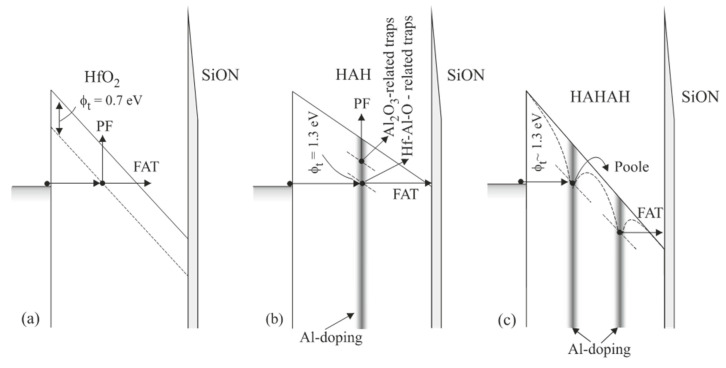
Schematic representation of the trap levels in different samples and corresponding dominant conduction mechanisms (**a**) HfO_2_ (75 cy); (**b**) HAH (36/5/36) and (**c**) HAHAH (24/2/24/2/24) (Reprinted with permission from [18]. Copyright 2015 American Chemical Society).

The energy location of the traps and how it is affected by Al introduction into HfO_2_ have been assessed by performing temperature-dependent I−V measurements and detailed analysis of conduction mechanisms. A comprehensive review of the approach to assessing important trap parameters from investigating dominant conduction mechanisms could be found in [36]. In Figure 4, the observed conduction mechanisms, and trap levels in line with the Al-doping profile in the different samples are schematically represented. It has been found [18] that in pure HfO_2_ the trap level is located at about 0.7 eV below the conduction band (Figure 4a), which is consistent with the theoretical calculations of the energy position of O-vacancies in HfO_2_ [26,37]. This level has not been observed in the Al-doped films, where traps have been estimated at 1.3 eV (Figure 4b,c). Similar results have also been observed by Molas et al. [38], who found a trap level at about 1.35 eV below the conduction band for HfAlO layers with Hf:Al(9:1) and at about 1.55 eV for Hf:Al(1:9). Therefore, the obtained results give evidence that doping with Al of HfO_2_ layers has two effects: (i) it reduces oxygen vacancies in HfO_2_, and (ii) introduces deep traps, which are involved in reversible trap processes. It will be shown below that these traps do not increase the leakage currents, and all Al-doped HfO_2_ layers have lower leakage currents than their HfO_2_ counterpart.

### 2.2. Composition of Charge Trapping Layer

**Figure 5:**
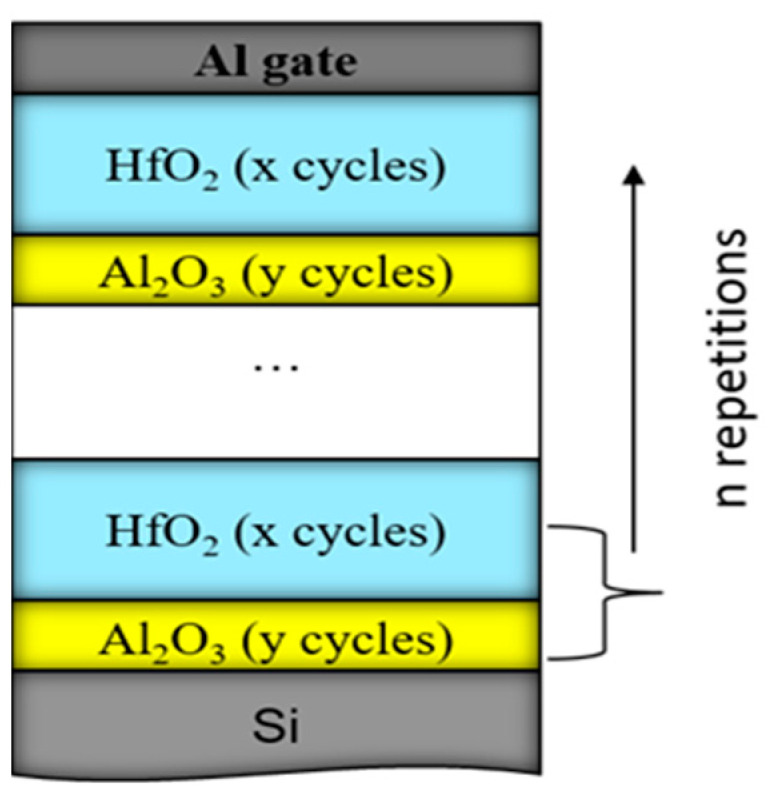
Schematic diagram of the HfO_2_/Al_2_O_3_ nanolaminated stacks. The used number of repetitions, n of the HfO_2_/Al_2_O_3_ bilayer structure is 5 or 10; HfO_2_ ALD cycles, x are 20 or 30, and Al_2_O_3_ ALD cycles, y, are 2, 5, 10 or 30. The overall stack thicknesses are varied between 15 nm and 65 nm.

Considering the results presented in Section 2.1, more detailed investigations on the CTL composition and how it affects charge trapping and storage characteristics have been performed [39]. Several nanolaminated dielectric structures have been prepared with different deposition cycles, respectively thickness, of HfO_2_ and Al_2_O_3_. Also, structures with different numbers of repetitions of the HfO_2_/Al_2_O_3_ bi-layer stack have been studied. For simplicity, the composition of the stack is designated as n×(x:y), where x is the number of HfO_2_ ALD deposition cycles, y—is the number of Al_2_O_3_ deposition cycles and n—is the number of repetitions of the HfO_2_/Al_2_O_3_ bi-layer stack (Figure 5). It should be mentioned that the deposition temperature is low—135 °C. More details for the deposition of samples can be found in [39]. These structures have no TO and BO to study the trapping efficiency of the charge trapping layer. Part of the samples have been subjected to RTA in O_2_ at 800 °C for 1 min. It should be mentioned that no crystallization of samples occurs after this annealing [40].

**Figure 6:**
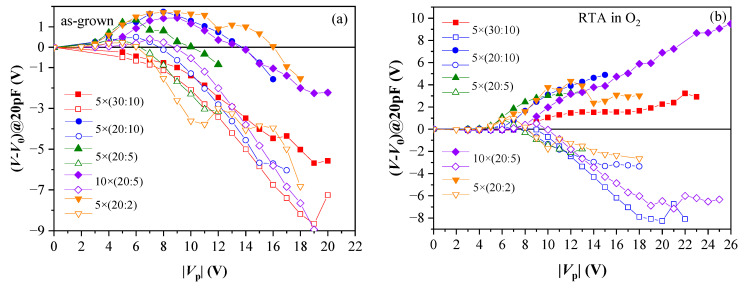
Evolution of C-V curve position at C = 20 pF with respect to its initial position V_0_ versus the voltage pulse magnitude: (**a**) as-grown samples; (**b**) RTA in O_2_. Solid symbols correspond to +V_p_; hollow to −V_p_. (Reprinted with permission from [39]. Copyright 2018 John Wiley and Sons).

Generally, the results have demonstrated that memory window is strongly affected by: annealing ambient, total thickness and Al_2_O_3_ content in the films. For as-deposited stacks, strong positive charge trapping is observed, while electron trapping is observed only at relatively low pulse voltages, V_p_ (Figure 6a). At higher voltages, positive charge build-up dominates even when electrons are injected into the stack. Such behaviour has been assigned to the existence of two competing processes—reversible electron trapping at existent traps and irreversible stress generation of positive charge; the latter outweighs electron trapping at higher V_p_. This result reveals that as-deposited samples are susceptible to high electric field stress. In addition, samples with the thickest HfO_2_ demonstrate the largest positive charge trapping, which is nearly unaffected by the Al_2_O_3_ amount in the films.

RTA in O_2_ substantially improves the memory window—stable electron trapping, which increases with increasing +V_p_ is observed (Figure 6b). It should be mentioned that no such effect has been observed after RTA in N_2_ at 800 °C [39]. Therefore, the increase in electron trapping is assigned to the impact of O_2_ than the high temperature. In addition, electron trapping is stronger in samples with more Al_2_O_3_ cycles; hence, the electron traps are related to the presence of Al atoms in HfO_2_, consistent with the results obtained in the previous section. It has been found that O_2_ annealing increases the density of electron traps but does not change their energy position, which has been estimated to lie at about 1.3 eV below CB of dielectric (also consistent with the results obtained in Section 2.1) [41]. After RTA in O_2_, positive charge trapping for all samples is weaker than before annealing and tends to saturation (Figure 6b). Therefore, it is concluded that RTA in O_2_ enhances the charge storage ability of the stacks and anneals defects in HfO_2,_ which are precursors of stress-induced positive charge. The number of Al_2_O_3_ deposition cycles is also very important—it should be small as the samples with thicker (30 cy) Al_2_O_3_ reveal strong degradation after O_2_ annealing [41].

**Figure 7:**
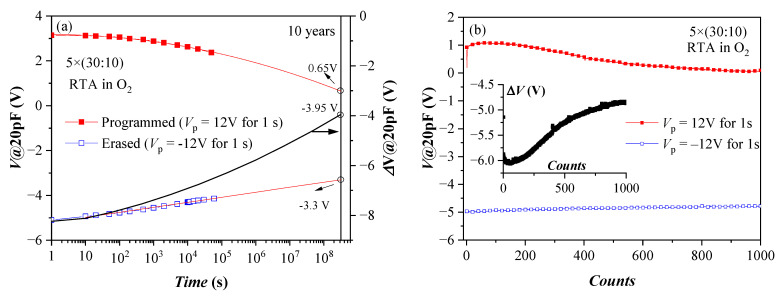
Retention (**a**) and endurance (**b**) characteristics of capacitors with 5×(30:10) stack annealed in O_2_. Solid lines in (**a**) represent linear fit of data in erased (after −V_p_) state and to c + pln^2^(t), where c and p are constants for the program (+V_p_) state. The black line (online colour) is the memory window evolution (right axis scale) (Reprinted with permission from [39]. Copyright 2018 John Wiley and Sons).

Retention characteristics of the annealed 5×(30:10) sample after a single P/E operation are presented in Figure 7a. These structures demonstrate good retention characteristics, considering they have no TO and BO. The approximation shows that more than 50% of the initial shift ΔV (hence stored charge) will be retained after ten years. The results also suggest that the negative charge (electrons) de-trapping rate is higher than that of the positive charge (holes). The detrapping of positive charge is well described by a logarithmic time dependence, which could be explained with the detrapping governed by tunnelling processes, that is, electrons/holes tunnel from the dielectric into the substrate (so-called first-order tunneling front model) [42,43]. The time dependence of negative charge loss is well described by a ln^2^(t) dependence. It was shown that using the simple model of a capacitor discharging through an impedance [44] based on Poole-Frenkel conduction leads to an expression close to the observed one. More rigorously, the square logarithm retention dependence was derived in [45]. Therefore, the charge loss mechanisms of electrons and holes in the stacks are different, which could be assigned to a different origin of the electron and hole charge traps. Stable endurance characteristics corresponding to positive charge build-up have been observed (Figure 7b). The negative charge trapping exhibits larger instabilities—the voltage shift resulting from electron trapping decreases with the number of cycles. The degradation of the memory window during the repeated write/erase operations is most likely due to wear-out mechanisms such as the generation of new bulk shallow traps and charges and interface state generation at the Si interface [46].

**Figure 8:**
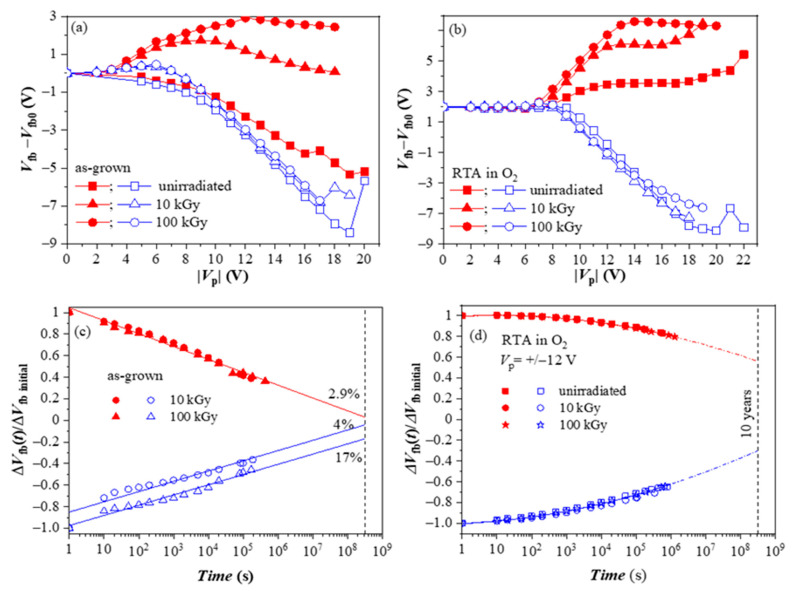
The evolution of the flat-band voltage changes on applying voltage pulses with different V_p_ and retention characteristics of the as-grown (**a**,**c**) and the O_2_ annealed stacks (**b**,**d**) before and after irradiation [47].

We have also investigated the radiation hardness of HfO_2_/Al_2_O_3_ CTL [47]. For this aim, the as-deposited and annealed 5×(30:10) stacks were subjected to ^60^Co γ -irradiation with two radiation doses (10 and 100 kGy). During irradiation, no bias was applied to the device. For both as-grown and O_2_-annealed samples, irradiation does not change the positive charge build-up behavior (Figure 8a,b). On the contrary, it significantly boosts electron trapping in both stacks. The stronger is the increase for as-deposited stacks. Based on these results, one may conclude that γ -irradiation is a viable way to increase the charge storage ability of the stacks. However, the investigation of retention characteristics revealed that despite the increased negative charge trapping, as-deposited stacks have poor retention (Figure 8c).

On the contrary, γ-radiation does not deteriorate the charge retention in oxygen-treated stacks (Figure 8d). The difference in the retention characteristics and the higher detrapping rate of the as-deposited stacks show evidence that radiation-induced traps have different natures than those produced by O_2_ annealing and are unsuitable for reliable storage. On the other hand, results demonstrate that the stacks after O_2_ annealing have good radiation tolerance to γ-rays up to very high doses of 100 kGy and can be successfully used in CTF devices working in a radiation-intensive environment.

### 2.3. Leakage Currents

**Figure 9:**
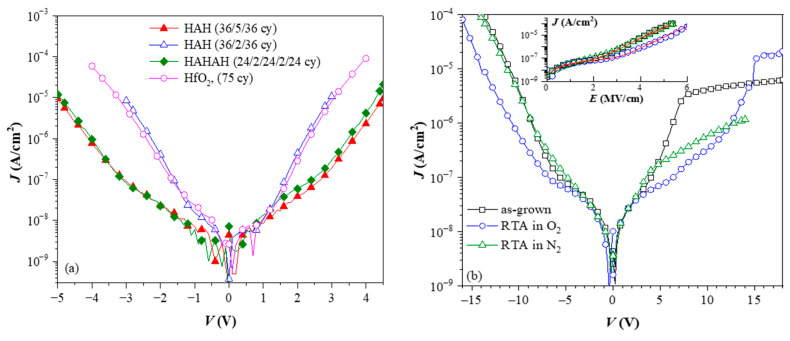
Leakage currents of pure HfO_2_ and three HfO_2_/Al_2_O_3_ stacks (**a**) (Reprinted with permission from [18]. Copyright 2015 American Chemical Society), and effect of thermal treatments in O_2_ and N_2_ on the current of 5×(30:10) stack (**b**) [47]. On (**a**), H denotes HfO_2_ sublayers and A− Al_2_O_3_ ones.

As mentioned above, the introduction of the Al in the stack does not deteriorate and even reduces the leakage currents for higher Al content in the films (Figure 9a). It has been established that oxygen annealing also decreases the leakage current of the stacks (Figure 9b). (Note that RTA in N_2_ (Figure 9b) does not improve the leakage current.) The reduction of leakage currents upon various oxygen annealing treatments is frequently reported for high-k materials. It is usually associated with the removal of oxygen vacancies accompanied by an elimination of the residuals (mainly carbon groups) from the precursors in the case of CVD and ALD processes. The decrease of the leakage current due to the Al-introduction could be related to some kind of band gap engineering, i.e., increasing the band gap of the stacks. However, the strong correlation between oxygen annealing, Al-content, and charge trapping suggests that there could be an alternative explanation for the leakage data—the leakage current reduction is most likely due to the effect of the trapped charges that modify the internal electric field of the stack. Since the introduction of Al into HfO_2_ creates specific trapping centres and oxygen treatment further enhances it, this would lead to leakage reduction.

## 3. Tunneling and Blocking Oxides

Blocking and tunnelling oxides are also important parts of the charge trapping stack. As mentioned above, in the current CTFs, SiO_2_ is used both as TO and BO. However, with the scaling of CTF cell dimensions, the thickness of TO and BO are also scaled-down, and the direct tunneling current through the thin tunnel SiO_2_ layer deteriorates the retention characteristics. High-k dielectrics are also considered to replace SiO_2_ as BO and TO. The use of material with a higher dielectric constant as a blocking layer ensures a lower electric field. Hence, carrier back-injection will be reduced. Substitution of tunnel SiO_2_ by the high-k dielectric enables the use of physically thicker TO, which can improve retention performance. However, the TO should also be trap-free to avoid trap-assisted tunnelling of the stored charges through the TO. This requirement is not easy to satisfy as the high-k dielectrics are trap-rich materials. Among the high-k dielectrics, Al_2_O_3_ has the largest band gap (more than 8 eV). Hence, the band-offsets with the CTL and Si will be the largest, which ensures more efficient storage in the quantum well formed by the tri-layer (BO-CTL-TO). Al_2_O_3_ also has good chemical and thermal stability and it is CMOS compatible. Several studies have shown that Al_2_O_3_ as BO improves the memory window, retention parameters and P/E efficiency and mitigates the problem of erase saturation [48,49,50]. Recently, all- AlO_x_ CTF stack in which BO, TO and CTL are engineered using different gas ratios and pulse times of the ALD process to obtain AlO_x_ layers with different thicknesses and oxygen content has been demonstrated [51].

**Figure 10:**
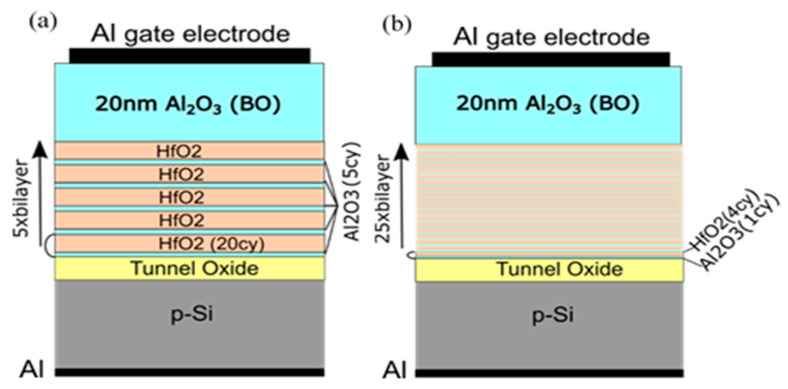
Schematics of the memory capacitors with nanolaminated HfO_2_/Al_2_O_3_ charge trapping stack (**a**) and Al–doped HfO_2_ charge trapping stack (**b**) [40].

**Figure 11:**
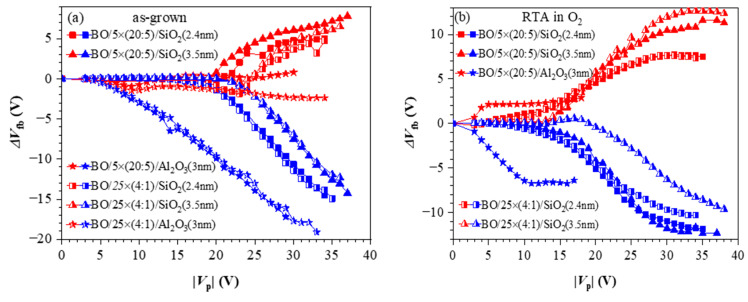
The flat band voltage shifts as a function of voltage pulse amplitude for different memory capacitor types: (**a**) before annealing; (**b**) after RTA in O_2_. Red symbols correspond to +V_p_ and the blue symbols to −V_p_ [40].

To investigate the influence of TO and BO on charge storage and reliability of CTF cells, we have prepared full charge trapping stacks consisting of CTL, tunnel and blocking oxides [40]. Two different CTLs were used in this case—nanolaminated stacks with 20 cy HfO_2_ and 5 cy Al_2_O_3_ repeated five times (5×(20:5)) (Figure 10a) and doped samples with 4 cy HfO_2_ and 1 cy Al_2_O_3_ repeated 25 times (25×(4:1)) (Figure 10b). As a tunnelling oxide, we used SiO_2_ with two thicknesses—2.4 and 3.5 nm, grown by standard thermal oxidation of Si. Stacks with 3 nm Al_2_O_3_ as TO, prepared by ALD, are also considered. As a blocking oxide, we used Al_2_O_3_ (about 20 nm) deposited under the same ALD conditions as those used for CTL and TO depositions. Al_2_O_3,_ as a tunnel and blocking oxide, enables the entire charge-trapping stack to be obtained in a single ALD deposition process, significantly simplifying the technology. The as-grown stacks with TO and BO, unlike stacks without TO and BO, demonstrate significant electron trapping. Hence, the memory window substantially increases (Figure 11a). It is seen that positive charge trapping depends on the tunnel oxide (and its thickness) and is weakly affected by the CTL.

On the contrary, the capture of electrons depends on the dielectric—it is stronger in the nanolaminated structures. It should be noticed that similarly to the as-deposited stacks without any TO and BO (Figure 6), the positive charge trapping increases progressively (almost linearly) with V_p,_ reaching very large values with no tendency for saturation. As discussed, such behaviour is explained by generating stress-induced positively charged defects. In structures with Al_2_O_3_ TO, regardless of the CTL, electron trapping is very weak, which makes them unsuitable as memory cells in CTF.

The impact of O_2_ annealing (Figure 11b) is very similar to that observed for the structures without TO and BO—the trapping of electrons increases significantly, and the positive charge trapping decreases and exhibits a saturation. In other words, these results confirm that after RTA in O_2,_ the stacks are more resistant to high-electric-field degradation and no positive charge is generated. Consequently, the net positive charge trapping decreases, the net negative charge trapping increases and the two branches of the trapping characteristics become more symmetrical. It should also be noted that compared to as-deposited stacks, the electron and hole trappings start at lower V_p_. Hence, the CTF can operate at lower voltages, which is one of the requirements the CTFs have to satisfy. The spatial density of trapped electrons, ρ_e_, and holes, ρ_h_, for various structures after RTA are calculated to be in the range ρ_h_ = (0.95–1.58) × 10^19^ cm^−3^ and ρ_e_ = (1.2–1.5) × 10^19^ cm^−3^ [40].

**Figure 12:**
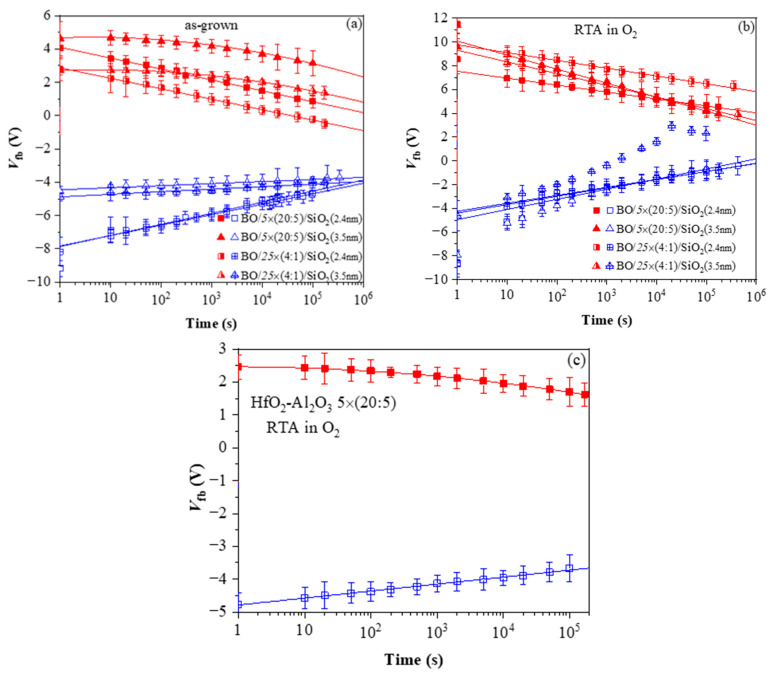
Charge retention characteristics in capacitors with various HfO_2_/Al_2_O_3_ stacks: (**a**) before and (**b**) after RTA in O_2_; (**c**) 5×(20:5) stack without BO and TO after RTA. The red symbols correspond to a negative charge (respectively, positive values of V_fb_) and the blue ones correspond to a positive charge (negative values of V_fb_) [40].

The retention characteristics of as-deposited stacks (Figure 12a) indicate that (i) the positive charge retention depends on the TO thickness and is independent of the dielectric stack; (ii) the discharge of positive charge follows a linear law which implies trap-to-band tunnelling mechanism; (iii) the discharge rate of positive charge is higher for stacks with thinner SiO_2_, while for 3.5 nm SiO_2,_ the discharge rate is very low, i.e., 3.5 nm SiO_2_ provides a good barrier to back-tunnelling of holes; (iv) the electron discharge follows different laws for the samples with 2.4 and 3.5 nm SiO_2_. For thinner TO, retention characteristics are linear. Hence, the discharge is performed via trap-to-band tunnelling. For the thicker TO, the characteristics are well fitted by ln^2^(t), which implies electron detrapping via the Poole–Frenkel mechanism; (v) the electron discharge curves of the two types of CTL are parallel to each other at an equal thickness of TO. Hence, the electron traps in the two kinds of charge trapping layers have the same origin, but their density is higher in multilayered 5×(20:5) stacks.

**Figure 13:**
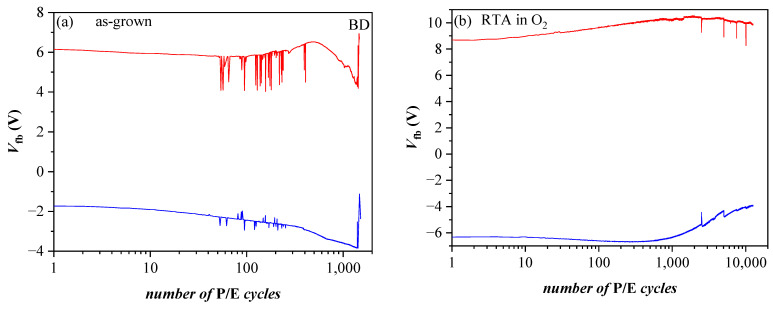
Endurance of BO/5×(20:5)/TO (3.5 nm SiO_2_) capacitors before (**a**) and after O_2_ annealing (**b**) measured under voltage pulses +/− 25 V. Red lines correspond to V_p_ = 25 V, blue ones to V_p_= −25 V [40].

Very significant changes in the discharge characteristics and their dependence on the parameters of the structure are provoked by O_2_ annealing (Figure 12b). It should be mentioned that these changes are unexpected. Generally, the retention characteristics of stacks are deteriorated after annealing. In addition, the electron discharge rate is higher in structures with a thicker 3.5 nm SiO_2_ than stacks with thinner 2.4 nm TO and slightly depends on the CTL. The discharge rate of holes is also higher after RTA and for thicker TO. Considering the obtained results [40] leads to the conclusion that the deteriorated characteristics are most likely due to a high-temperature-induced interaction between the HfO_2_/Al_2_O_3_ charge trapping layer and the TO and the formation of defects due to this interaction. These defects, located at the CTL/TO interface and/or in the TO itself, cause a faster discharge of the charges stored in the CTL [52]. Defects generated by the annealing in the Al_2_O_3_ blocking oxide as a possible leakage path could not be rejected as well. As commented in [51], in Al_2_O_3_ deposited by ALD with H_2_O as oxidant, different species such as Al-OH, Al-O-H, Al-Al could be formed, resulting in increased density of defects. In [41], we have demonstrated that annealing in O_2_ creates different electrically active defects depending on the Al_2_O_3_ thickness in HfO_2_/Al_2_O_3_ stacks. In the case of thick (30 cy) Al_2_O_3_, the generation of a negative charge has been observed, accompanied by a substantial increase in leakage current. The retention characteristics of the annealed 5×(20:5) stacks without any intentionally grown TO and BO (Figure 12c) support this conclusion—they are very similar to the retention in stacks with a thicker SiO_2_ TO and BO before annealing. The endurance characteristics (Figure 13a) before annealing reveal instabilities, especially in electron trapping. Substantial degradation and progressive accumulation of positive charge have been observed for P/E cycles > 600. This supports the conclusion that the structures before RTA are vulnerable to high electric field stress degradation. After RTA in O_2,_ the structures demonstrate better endurance and can withstand more than 10^4^ P/E cycles without coming to breakdown (BD) (Figure 13b).

**Figure 14:**
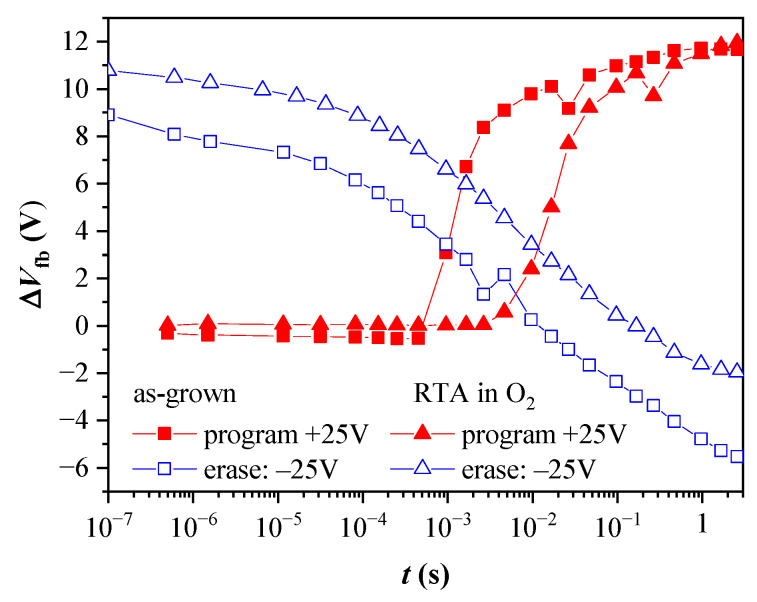
Dependence of the flat band voltage shifts for program and erase operations on the voltage pulse width, *t*_p_ for capacitors with BO and 2.4 nm TO, before and after annealing. The programming voltage is +25 V, and the erasing is −25 V.

The obtained program and erase speeds are illustrated in Figure 14 for capacitors with 2.4 nm TO before and after oxygen annealing. The capacitors exhibit almost negligible electron trapping at pulses shorter than 10^−4^ s for the as-grown samples and 10^−3^ s for the annealed ones. In both cases, after the threshold pulse time, the electron accumulation in the CTL is rapid. The increase of pulse duration, t_p,_ within one decade results in the accumulation of more than 70% of the stored negative charge measured at t_p_ = 1 s. (The overall shape of the dependence ΔV_fb_ vs. t_p_ before and after annealing is the same—steep increase followed by a gradual increment with a tendency of saturation for t_p_ > 1 s). The detrapping of the captured electrons under negative V_p_ does not show abrupt change with the value of t_p_. The more efficient electron release is observed at t_p_ above 10^−5^ s, and the full discharge state is reached at ~10^−2^ s and 10^−1^ s, for the as-grown and annealed stacks, respectively. The accumulation of positive charge in the CTL (“over-erasing”) requires pulse times about 100 times higher than the ones for the electron trapping under the same V_p_ magnitude. Hence, it can be concluded that the annealing increases the pulse duration (~10 times) needed to program the capacitor and return it to its initial state. This result agrees with the degradation of the retention characteristics after annealing and could be related to the defect generation in both the TO and BO layers. However, we should mention that the program/erase speeds obtained with a capacitor type of structure could be affected by the availability of inversion carriers in the Si substrate. As demonstrated in [53], the inversion current of tunnel MOS capacitors on p-type substrates is dominated by the thermal generation rate of minority electrons via traps at the Si/SiO_2_ interface and in the deep depletion region. Since the thermal generation at room temperature is slow, the measurements are conducted under illumination to neutralize this effect.

It is helpful to compare the obtained results with the performance of conventional CT memory cells with the ONO stack. Ramkumar [15] reported very good endurance characteristics of poly-Si/oxide/nitride/oxide/Si (SONOS) cells with very small shifts of threshold voltage after 10^6^ P/E cycles. The retention in the erase state is also very good. However, in the program state at 85 °C, a significant loss of stored charge is observed (more than 50% at ten years). Similar stable retention performance in the erase state and faster detrapping rate in the program state demonstrate our stacks in Figure 12a. In Table 1, memory windows, program speeds and the time needed to reach the full window of ONO stacks reported in different works are given. 

The memory windows of ONO structures are smaller than those of our HfO_2_/Al_2_O_3_ stacks. The program speed of ONO stacks, however, is better—10^−5^ s. It should be mentioned that the excellent program speed of cylindrical cells with ONO stacks reported in [59,60] (Table 1) is due to hyperbolic dependence of the electric field along the radial coordinate, which enhances the field at the TO interface while decreasing it at BO interface. In other words, programming speed depends strongly on the device geometry as well as on how the carriers are injected (faster speeds are achieved in the case of hot carrier injection (in the order of µs) as compared to ms in the case of Fowler-Nordheim tunnelling [15] also used in our samples). As mentioned, scaling rules and integration compatibility with CMOS process flow require the replacement of SONOS cells with MOHOS. Many works study charge trapping and storage in different MOHOS structures. Comparison of their properties could be considered only qualitatively because all performance characteristics depend very strongly on the materials used for the different layers in the charge trapping stack and the technology (including deposition technique, deposition parameters and annealing steps). For example, as shown by Agrawal et al. [51], even changing parameters of the deposition process (gas flow ratio and pulse deposition time of precursors) results in Al-oxide layers with substantially different properties in this way allowing the engineering of band-gap of the layer. What concerns ALD, the most widely used technology for high-quality quality, very thin high-k dielectric layers, the precursors used for the deposition process are also of utmost importance. Here, we will compare our results with results obtained on similar HfO_2_/Al_2_O_3_ stacks prepared by ALD. Consistent with our work, the memory windows reported by other authors [61,62] for CT-NVM with HfO_2_/Al_2_O_3_ CTL are substantially larger (8–12 V) than those of ONO stacks. The work of Yoo, et al. [61] confirms the enhancement of the memory window after O_2_ annealing. Very good retention of more than 76% at ten years is reported in [62] for samples with optimized Hf:Al ratio. In this work, it is also demonstrated by XPS that Al incorporation reduces oxygen vacancies in HfO_2_—the result is also inferred from our consideration of I-V characteristics of Al-doped HfO_2_ (Section 2.1).

All- AlO_x_ CT stacks reported in [51] reveal better retention than the respective stacks with SiN_x_ CTL, further improved by a low-temperature N_2_ annealing. These stacks also demonstrate stable erase retention and faster discharge in the program state, consistent with our results and results reported in [15] for ONO. Hou et al. [63] reported very good charge trapping and storage characteristics of HfO_2_/Al_2_O_3_ CTL, where better performance is obtained for structures annealed at 1000 °C in N_2._ This result is assigned to better intermixing between HfO_2_ and Al_2_O_3_ at high temperatures and the formation of Hf-rich HfAlO nanocrystals. These authors also suggested [50] short O_3_ treatment of Si to obtain better Al_2_O_3_/Si interfacial properties. Improvement of retention by the engineering of tunnelling oxide is suggested in the work of Song et al. [64]. They demonstrated by using Synopsis simulations that incorporation of Al_2_O_3_ in the tunnelling oxide (i.e., SiO_2_/Al_2_O_3_/SiO_2_ stack) results in a significant improvement in retention. All the investigations reported by other authors have been performed on stacks deposited at substantially higher temperatures (250–350 °C) compared to 135 °C, which is used for the deposition of our samples. This low-temperature deposition may be the reason for increased defect density and insufficient performance of Al_2_O_3_ as blocking oxide in our samples. Therefore, an increase in the deposition temperature of Al_2_O_3_ BO is a possible way to improve its insulating properties.

## 4. Conclusions and Perspectives

We have demonstrated that the charge trapping ability of HfO_2_/Al_2_O_3_ charge trapping layers could be tailored and enhanced by optimization of stack parameters as well as annealing steps. Aluminium introduces deep traps with high density without compromising the leakage currents. Electron trapping in HfO_2_/Al_2_O_3_ stacks is also substantially increased by O_2_ annealing. On the other hand, both Al introduction and O_2_ annealing reduce oxygen vacancies in HfO_2,_ thus decreasing the density of shallow traps. They also improve the vulnerability of the stacks to high electric field stress. Therefore, the combination of Al-introduction in HfO_2_ and O_2_ annealing resulted in improved trapping and storage ability, which manifested as large memory windows and good retention and endurance of CT stacks without any tunnel and blocking layers. The introduction of BO and TO, quite unexpectedly, turns out to be very challenging. Despite the increased electron trapping and improved susceptibility to high-field stress, the structures after high-temperature O_2_ annealing demonstrate degraded retention characteristics and increased program time (due most likely to defects generated in the blocking Al_2_O_3_ and the interfacial reaction between CTL and TO). The retention of stacks with TO and BO before annealing is good, but their endurance is compromised by the high electric field vulnerability of HfO_2_/Al_2_O_3_ stacks without O_2_ annealing.

**Figure 15:**
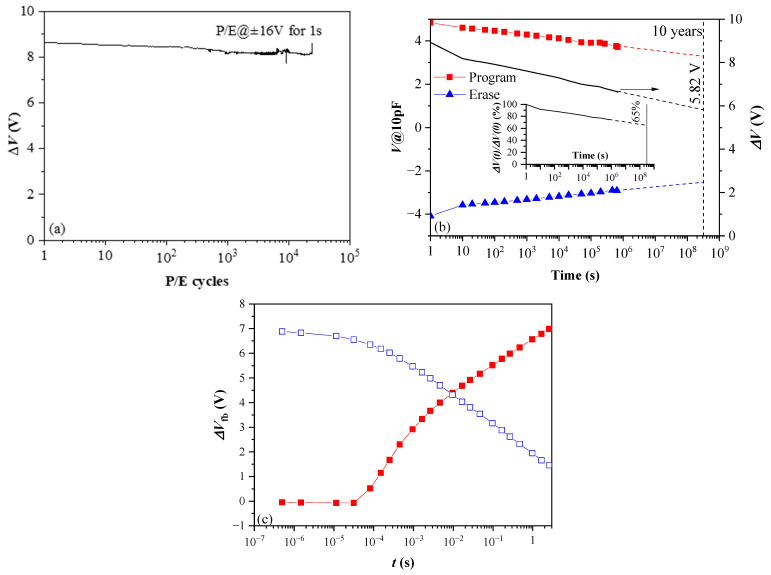
Endurance (**a**) retention (**b**) and programming/erase speed of 10×(30:10) HfO_2_/Al_2_O_3_ nanolaminated CTL layer furnace annealed in O_2_ at 600 °C (Reprinted with permission from [41]. Copyright 2018 IOP Publishing Ltd.); (**c**) V_fb_ shifts during program (red closed symbols) and erase (blue, open symbols) operation with pulses with incremental durations.

From this point of view, the results (Figure 15) obtained for thicker 10×(30:10) samples without BO and TO, which were subjected to standard thermal annealing in O_2_ at lower (600 °C) temperature [41], give some promise. As is seen, the retention and endurance characteristics are significantly better than that of the as-deposited samples with TO and BO (Figure 15a)—more than 75% of charge is retained after 10^6^ s and the linear extrapolation shows that after ten years, a significant amount of charge (65%) will still be stored. The structures also show good endurance—ΔV changes by ~6% after 2.5 × 10^4^ P/E cycles (Figure 15b). In addition, the program speed is faster than an order of magnitude (Figure 15c). (However, we have to note that apart from technological conditions and stack geometry, better programming speed can also be influenced by the initial fixed oxide charge, which is positive with high density for the stack in Figure 15c and negative for stacks in Figure 14, i.e., more detailed investigation is needed). Therefore, the decrease of annealing temperature could be a feasible way toward further optimization and improvement of the performance of CTF cells. Even more fascinating is the idea to design a CTF cell without BO and TO. A CTF without TO and BO is reported in [65] and is considered a viable way for designing a new class of scalable flash memory devices. The device reported in [65] is realized with aluminium oxide phosphate dielectric deposited by spin-coating and processed at low (<200 °C) temperature. Our 10×(30:10) stacks outperform in most parameters the one reported in [65].

Another technology approach that could benefit from the implementation of HfO_2_/Al_2_O_3_ CTL stacks is the recently suggested hybrid CT memories, which combine CT functionality with the ferroelectric (FE)/antiferroelectric (AFE) properties of HfO_2_. In this case, combinations of dielectric, FE and AFE variations of HfO_2_ are used to tailor voltage distribution across the stack (e.g., enforcing a large electric field on a tunnel barrier and reducing the internal field in CTL). FE/AFE HfO_2_-based layer could be introduced as a part of CTL or even as a BO, and the modulation of an electric field is achieved by the dipole switching, which results in an enhancement of performance characteristics—increased switching speed and robust retention and endurance [66,67]. These devices take advantage of the faster polarization switching mechanisms compared to electron injection through TO, which results in increased P/E speed. However, the technology of these multi-layered stacks comprising FE/AFE states is more complicated, and the thermal budget is much higher as it involves high-temperature steps for the crystallization of films.

In the present work, we focused on HfO_2_/Al_2_O_3_ stacks from the viewpoint of their implementation in charge-trapping flash memories. It should be mentioned that these stacks are also intensively investigated as a functional part of other computational and memory concepts. The first to mention is the resistive random-access memories (RRAM), which have emerged as one of the most promising alternatives for future NVMs. Moreover, RRAM devices can demonstrate neuronal dynamics. Hence, they are considered for neuromorphic computational applications. RRAM outperforms transistor-type memories regarding high-speed operation, low power consumption and high integration density. Recently, multi-level switching and synaptic behaviour of HfO_2_/Al_2_O_3_ stacks have been reported in several papers [68,69,70,71]. The enhanced switching performance of these stacks is assigned to the migration of oxygen vacancies due to their different density in HfO_2_ and Al_2_O_3_ layers [72,73].

In recent years, flexible electronics have become a special interest because of their importance for wearable health management devices, flexible displays, sensors and even artificial skin and soft robotics. The possibility to deposit HfO_2_-based high-k dielectrics at relatively low temperatures by ALD opens up new horizons for manufacturing devices with high stability and mechanical resilience on flexible substrates for application in flexible electronics [74,75]. In this regard, thin film transistors (TFT) with active channel material of InGaZnO (IGZO) are considered especially promising [76,77]. It has been shown that HfO_2_/Al_2_O_3_ stacks can boost the performance of IGZO-based TFTs [78]. Integration of HfO_2_/Al_2_O_3_ stacks in recently reported synaptic transistors based on IGZO nanofibers [79,80] could be a feasible way to enhance their retention characteristics. It could open new opportunities to realize bio-inspired in-memory computing. Very promising electrical characteristics of bendable and biodegradable metal-oxide-semiconductor field-effect transistors and capacitors fabricated by integrating HfO_2_/Al_2_O_3_ high-k bilayers on silicon nanomembranes (Si NMs) and utilizing polymeric substrates have been reported in [81]. This demonstrates the potential of these stacks to be utilized in on-demand water-soluble Si NM-based devices toward futuristic applications in disposable electronics and temporary biomedical implants.

## Figures and Tables

**Table 1:** Comparison of memory windows, program speeds and time to reach the full window of ONO stacks reported in different works.

Memory Window, V	Program Speed, s	Time to Full Window, s	Reference
5.5	10^−5^	10^−1^	[54]
2.5	3 × 10^−5^	10^−1^	[55]
5.5	10^−5^	10^−1^	[56]
8, on c-Si 10.7, on poly-Si	-	-	[57]
6	10^−5^	10^−1^	[58]
4 cylindrical cell	10^−7^	10^−3^	[59,60]

## Data Availability

The data presented in this study are available on request from the corresponding author.

## References

[1] Yinug F. (2007). The Rise of the Flash Memory Market: Its Impact on Firm Behavior and Global Semiconductor Trade Patterns. J. Int. Commer. Econ..

[2] Patel D. The History and Timeline of Flash Memory. https://www.semianalysis.com/p/the-history-and-timeline-of-flash.

[3] The Statistic Portal. 2013–2021. https://www.statista.com.

[4] Yoshimitsu Y., Yoshinari K., Toshimasa M. (2013). Scalable Virtual-Ground Multilevel-Cell Floating-Gate Flash Memory. IEEE Trans. Electron. Devices.

[5] Goda A. (2021). Recent Progress on 3D NAND Flash Technologies. Electronics.

[6] Kahng D., Sze S.M. (1967). A Floating Gate and Its Application to Memory Devices. Bell Syst. Tech. J..

[7] Chen A. (2016). A review of emerging non-volatile memory (NVM) technologies and applications. Solid-State Electron..

[8] International Roadmap for Devices and Systems (IRDS™) 2021 Edition. https://irds.ieee.org/editions/2021.

[9] Rosmeulen M., Van Houdt J. NAND Flash: The Industry’s Workhorse for Data Storage Applications. IMEC. https://www.imec-int.com/en/articles/role-3d-nand-flash-and-fefet-data-storage-roadmap.

[10] Dimitrakis P., Dimitrakis P. (2015). Charge-Trapping Non-Volatile Memories.

[11] Zhao C., Zhao C.Z., Taylor S., Chalker P.R. (2014). Review on Non-Volatile Memory with High-k Dielectrics: Flash for Generation Beyond 32 nm. Materials.

[12] Park G.H., Cho W.J. (2010). Reliability of modified tunneling barriers for high performance nonvolatile charge trap flash memory application. Appl. Phys. Lett..

[13] Wegner H.A.R., Lincoln A.J., Pao H.C., O’Connel M.R., Oleksiak R.E., Lawrence H. (1967). The variable threshold transistor, a new electrically-alterable, non-destructive read-only storage device. IEDM Tech. Dig..

[14] Hwang C.S. (2015). Prospective of Semiconductor Memory Devices: From Memory System to Materials. Adv. Electron. Mater..

[15] Ramkumar K., Dimitrakis P., Valov I., Tappertzhofen S. (2022). Charge trapping NVMs with metal oxides in the memory stack. Metal Oxides for Non-Volatile Memory: Materials, Technology and Application.

[16] Park P.K., Kang S.W. (2006). Enhancement of dielectric constant in HfO_2_ thin films by the addition of Al_2_O_3_. Appl. Phys. Lett..

[17] You H.W., Cho W.J. (2010). Charge trapping properties of the HfO_2_ layer with various thicknesses for charge trap flash memory applications. Appl. Phys. Lett..

[18] Paskaleva A., Rommel M., Hutzler A., Spassov D., Bauer A.J. (2015). Tailoring the electrical properties of HfO_2_ MOS-devices by aluminum doping. ACS Appl. Mater. Interfaces.

[19] Spiga S., Driussi F., Lamperti A., Congedo G., Salicio O. (2012). Effects of Thermal Treatments on the Trapping Properties of HfO_2_ Films for Charge Trap Memories. Appl. Phys. Express.

[20] Kim J., Kim J., Cho E.C., Yi J. (2021). Analysis of HfO_2_ Charge Trapping Layer Characteristics After UV Treatment. ECS J. Solid State Sci. Technol..

[21] Zhu C., Huo Z., Xu Z., Zhang M., Wang Q., Liu J., Long S., Liu M. (2010). Performance Enhancement of Multilevel Cell Nonvolatile Memory by Using a Bandgap Engineered High-k Trapping Layer. Appl. Phys. Lett..

[22] Lan X., Ou X., Cao Y., Tang S., Gong C., Xu B., Xia Y., Yin J., Li A., Yan F. (2013). The effect of thermal treatment induced inter-diffusion at the interfaces on the charge trapping performance of HfO_2_/Al_2_O_3_ nanolaminate- based memory devices. J. Appl. Phys..

[23] Cui Z., Xin D., Kim T., Choi J., Cho J., Yi J. (2021). Improvement of the Charge Retention of a Non-Volatile memory by a Bandgap-Engineered Charge Trap Layer. ECS J. Solid State Sci. Technol..

[24] Hou X., Yan X., Liu C., Ding S., Zhang D.W., Zhou P. (2018). Operation mode switchable charge-trap memory based on few-layer MoS_2_. Semicond. Sci. Technol..

[25] Paskaleva A., Lemberger M., Bauer A.J., Weinreich W., Heitmann J., Erben E., Schröder U., Oberbeck L. (2009). Influence of the amorphous/crystalline phase of Zr_1-x_Al_x_O_2_ high-k layers on the capacitance performance of metal insulator metal stacks. J. Appl. Phys..

[26] Mannequin C., Gonon P., Vallée C., Latu-Romain L., Bsiesy A., Grampeix H., Salaün A., Jousseaume V. (2012). Stress-Induced Leakage Current and Trap Generation in HfO_2_ Thin Films. J. Appl. Phys..

[27] De Salvo B., Ghibaudo G., Pananakakis G., Guillaumot B., Reimbold G. (2000). A General Bulk-Limited Transport Analysis of a 10 nm-Thick Oxide Stress-Induced Leakage Current. Solid-State Electron..

[28] Hou Z.Z., Wang G.L., Xiang J.J., Yao J.X., Wu Z.H., Zhang Q.Z., Yin H.X. (2017). Improved Operation Characteristics for Nonvolatile Charge-Trapping Memory Capacitors with High-κ Dielectrics and SiGe Epitaxial Substrates. Chin. Phys. Lett..

[29] Suh D.C., Cho Y.D., Kim S.W., Ko D.H., Lee Y., Cho M.H., Oh J. (2010). Improved thermal stability of Al_2_O_3_/HfO_2_/Al_2_O_3_ high-k gate dielectric stack on GaAs. Appl. Phys. Lett..

[30] Byun Y.C., Mahata C., An C.H., Kim H. (2012). Starting layer dependence of the atomic-layer-deposited HfAlO_x_ films on GaAs. Semicond. Sci. Technol..

[31] An C.H., Mahata C., Byun Y.C., Kim H.J. (2013). Atomic-layer-deposited (HfO_2_)_1−x_(Al_2_O_3_)_x_ nanolaminate films on InP with different Al_2_O_3_ contents. J. Phys. D Appl. Phys..

[32] Kiani A., Bayer B.C., Hasko D.G., Milne W.I., Flewitt A.J. (2017). Analysis of amorphous indium-gallium-zinc-oxide thin-film transistors with bi-layer gate dielectric stacks using maxwell-wagner instability model. ECS Trans..

[33] Fenag Q., Yan F., Luo W., Wang K. (2016). Charge trap memory based on few-layer black phosphorus. Nanoscale.

[34] Zhang E., Wang W., Zhang C., Jin Y., Zhu G., Sun Q., Zhang D.W., Zhou P., Xiu F. (2015). Tunable charge-trap memory based on few-layer MoS_2_. ACS Nano.

[35] Baik S.J., Hyunjung S. (2021). Charge Trapping in Amorphous Dielectrics for Secure Charge Storage. ACS Appl. Mater. Interfaces.

[36] Paskaleva A., Spassov D., Danković D. (2017). Consideration of conduction mechanisms in high-k dielectric stacks as a tool to study electrically active defects. Facta Univ. Ser. Electron. Energetics.

[37] Gavartin J.L., Muñoz Ramo D., Shluger A.L., Bersuker G., Lee B.H. (2009). Negative oxygen vacancies in HfO_2_ as charge traps in high-k stacks. Appl. Phys. Lett..

[38] Molas G., Bocquet M., Buckley J., Grampeix H., Gély M., Colonna J.-P., Licitra C., Rochat N., Veyront T., Garros X. (2007). Investigation of hafnium-aluminate alloys in view of integration as interpoly dielectrics of future flash memories. Solid State Electron..

[39] Spassov D., Paskaleva A., Krajewski T.A., Guziewicz E., Luka G., Ivanov T. (2018). Al_2_O_3_/HfO_2_ multilayer high-k dielectric stacks for charge trapping flash memories. Phys. Status Solidi A.

[40] Spassov D., Paskaleva A., Guziewicz E., Wozniak W., Stanchev T., Ivanov T., Wojewoda-Budka J., Janusz-Skuza M. (2022). Charge storage and reliability characteristics of nonvolatile memory capacitors with HfO_2_/Al_2_O_3_-based charge trapping layers. Materials.

[41] Spassov D., Paskaleva A., Krajewski T.A., Guziewicz E., Luka G. (2018). Hole and electron trapping in HfO_2_/Al_2_O_3_ nanolaminated stacks for emerging non-volatile flash memories. Nanotechnology.

[42] McWhorter P.J., Miller S.L., Miller W.M. (1990). Modeling the anneal of radiation-induced trapped holes in a varying thermal environment. IEEE Trans. Nucl. Sci..

[43] Kamohara S., Okumura T. (2008). New physical model to explain logarithmic time dependence of data retention in flash EEPROM. Appl. Surf. Sci..

[44] Chen J.J., Mielke N.R., Hu C.C., Tewksbury S.K., Brewer J.E. (2007). Flash memory reliability. Nonvolatile Memory Technologies with Emphasis on Flash: A Comprehensive Guide to Understanding and Using NVM Devices.

[45] Lehovec K., Fedotowsky A. (1978). Charge retention of MNOS devices limited by Frenkel Poole detrapping. Appl. Phys. Lett..

[46] Grossi A., Zambelli C., Olivo P., Micheloni R. (2016). Reliability of 3D NAND Flash Memories. 3D Flash Memories.

[47] Spassov D., Paskaleva A., Guziewicz E., Davidović V., Stanković S., Djorić-Veljković S., Ivanov T., Stanchev T., Stojadinović N. (2021). Radiation tolerance and charge trapping enhancement of ALD HfO_2_/Al_2_O_3_ nanolaminated dielectrics. Materials.

[48] Lee C.H., Hur S.H., Shin Y.C., Choi J.H., Park D.G., Kim K. (2005). Charge-trapping device structure of SiO_2_/SiN/high-k dielectric Al_2_O_3_ for high-density flash memory. Appl. Phys. Lett..

[49] Seo Y.J., Kim K.C., Kim H.D., Joo M.S., An H.M., Kim T.G. (2008). Correlation between charge trap distribution and memory characteristics in metal/oxide/nitride/oxide/silicon devices with two different blocking oxides, Al_2_O_3_ and SiO_2_. Appl. Phys. Lett..

[50] Hou Z., Wu Z., Yin H. (2018). Performance enhancement for charge trapping memory by using Al_2_O_3_/HfO_2_/Al_2_O_3_ tri-layer high-κ dielectrics and high work function metal gate. ECS J. Solid State Sci. Technol..

[51] Agrawal K., Yoon G., Kim J., Chavan G., Kim J., Park J., Phong P., Cho E., Yi J. (2020). Improving Retention Properties of ALD-Al_x_O_y_ Charge trapping layer for non-volatile memory application. ECS J. Solid State Sci. Technol..

[52] Arreghini A., Zahid M.B., Van den Bosch G., Suhane A., Breuil L., Cacciato A., Van Houdt J. (2011). Effect of high temperature annealing on tunnel oxide properties in TANOS devices. Microelectron. Eng..

[53] Lin C.-H., Hsu B.-C., Lee M.H., Liu C.W. (2001). A Comprehensive Study of Inversion Current in MOS Tunneling Diodes. IEEE Trans. Electron. Dev..

[54] Ramkumar K., Prabhakar V., Kapre R. Scalable SONOS Based Embedded Non-Volatile Memory Technology. https://sst.semiconductor-digest.com/.

[55] Seo Y.J., An H.M., Kim H.D., Kim T.G. (2009). Improved Performance in Charge-Trap-Type Flash Memories with an Al_2_O_3_ Dielectric by Using Bandgap Engineering of Charge-Trapping Layers. J. Korean Phys. Soc..

[56] Chen T.-S., Wu K.-H., Chung H., Kao C.-H. (2004). Performance Improvement of SONOS Memory by Bandgap Engineering of Charge-Trapping Layer. IEEE Electr. Dev. Lett..

[57] Jeong J.-K., Sung J.-Y., Ko W.-S., Nam K.-R., Lee H.-D., Lee G.-W. (2021). Physical and Electrical Analysis of Poly-Si Channel Effect on SONOS Flash Memory. Micromachines.

[58] Vianello E., Driussi F., Arreghini A., Palestri P., Esseni D., Selmi L., Akil N.J., van Duuren M., Golubović D.S. (2009). Experimental and Simulation Analysis of Program/Retention Transients in Silicon Nitride-Based NVM Cells. IEEE Trans. Electr. Dev..

[59] Fu J., Singh N., Buddharaju K.D., Teo S.H., Shen C., Jiang Y., Zhu C.X., Yu M.B., Lo G.Q., Balasubramanian N. (2008). Si-nanowire based gate-all-around nonvolatile SONOS memory cell. IEEE Electron. Dev. Lett..

[60] Gnani E., Reggiani S., Gnudi A., Baccarani G., Fu J., Singh N., Lo G.Q., Kwong D.L. (2010). Modeling of gate-all-around charge trapping SONOS memory cells. Solid-State Electron..

[61] Yoo J., Kim S., Jeon W., Park A., Choi D., Choi B. (2019). A Study on the Charge Trapping Characteristics of High-k Laminated Traps. IEEE Electron. Device Lett..

[62] Yoon G., Kim T., Agrawal K., Kim J., Park J., Kim H.H., Cho E.C., Yi J. (2020). Optimization of MIS type Non-volatile Memory Device with Al-doped HfO_2_ as Charge Trapping Layer. ECS J. Solid State Sci. Technol..

[63] Hou Z., Wu Z., Yin H. (2018). The Effect of Thermal Treatment Induced Performance Improvement for Charge Trapping Memory with Al_2_O_3_/(HfO_2_)0.9(Al_2_O_3_)0.1/Al_2_O_3_ Multilayer Structure. ECS J. Solid State Sci. Technol..

[64] Song Y.S., Park B.-G. (2021). Retention Enhancement in Low Power NOR Flash Array with High-κ–Based Charge-Trapping Memory by Utilizing High Permittivity and High Bandgap of Aluminum Oxide. Micromachines.

[65] Mondal S., Venkataraman V. (2019). Low temperature below 200 °C solution processed tunable flash memory device without tunneling and blocking layer. Nat. Commun..

[66] Ali T., Mertens K., Olivo R., Rudolph M., Oehler S., Kühnel K., Lehninger D., Müller F., Lederer M., Hoffmann R. A novel hybrid high-speed and low power antiferroelectric hso boosted charge trap memory for high-density storage. Proceedings of the IEEE International Electron Devices Meeting (IEDM).

[67] Shin E.J., Shin S.W., Lee S.H., Lee T.I., Kim M.J., Ahn H.J., Kim J.H., Hwang W.S., Lee J., Cho B.J. Capacitance boosting by anti-ferroelectric blocking layer in charge trap flash memory device. Proceedings of the IEEE International Electron Devices Meeting (IEDM).

[68] Wang Q., Wang Y., Luo R., Wang J., Ji L., Jiang Z., Wenger C.H., Song Z., Song S., Ren W. (2022). Ultrathin HfO_2_/Al_2_O_3_ bilayer based reliable 1T1R RRAM electronic synapses with low power consumption for neuromorphic computing. Neuromorphic Comput. Eng..

[69] Huang X., Wu H., Gao B., Sekar D.C., Dai L., Kellam M., Bronner G., Deng N., Qian H. (2016). HfO_2_/Al_2_O_3_ multilayer for RRAM arrays: A technique to improve tail-bit retention. Nanotechnology.

[70] Basnet P., Anderson E.C., Athena F.F., Chakrabarti B., West M.P., Vogel E.M. (2023). Asymmetric Resistive Switching of Bilayer HfO_x_/AlO_y_ and AlO_y_/HfO_x_ Memristors: The Oxide Layer Characteristics and Performance Optimization for Digital Set and Analog Reset Switching. ACS Appl. Electron. Mater..

[71] Kim S., Chen J., Chen Y.C., Kim M.H., Kim H., Kwon M.W., Hwang S., Ismail M., Li Y., Miao X.S. (2019). Neuronal dynamics in HfO_x_/AlO_y_ based homeothermic synaptic memristors with low power and homogeneous resistive switching. Nanoscale.

[72] Liu J., Yang H., Ma Z., Chen K., Zhang X., Huang X., Oda S. (2017). Characteristics of multilevel storage and switching dynamics in resistive switching cell of Al_2_O_3_/HfO_2_/Al_2_O_3_ sandwich structure. J. Appl. Phys. D.

[73] Khera E.A., Mahata C., Imran M., Niaz N.A., Hussain F., Arif Khalil R.M., Rasheed U., Kim S. (2022). Improved resistive switching characteristics of a multi-stacked HfO_2_/Al_2_O_3_/HfO_2_ RRAM structure for neuromorphic and synaptic applications: Experimental and computational study. RSC Adv..

[74] Rodriguez-Lopez O., Ruiz E.G., Polednik A.J., Duran-Martinez A.C., Garcia-Sandoval A., Voit W., Gutierrez-Heredia G. (2021). Electrical characterization of flexible hafnium oxide capacitors on deformable softening polymer substrate. Microel. Eng..

[75] Wang B., Huang W., Chi L., Al-Hashimi M., Marks T.J., Facchetti A. (2018). High-k gate dielectrics for emerging flexible and stretchable electronics. Chem. Rev..

[76] Barquinha P., Pereira L., Gonçalves G., Martins R., Fortunato E. (2009). Toward high-performance amorphous GIZO TFTs. J. Electrochem. Soc..

[77] Yang J., Yang X., Zhang Y., Che B., Ding X., Zhang J. (2019). Improved gate bias stressing stability of IGZO thin flm transistors using high-k compounded ZrO_2_/HfO_2_ nanolaminate as gate dielectric. Mol. Cryst. Liq. Cryst..

[78] Shi Q., Aziz I., Ciou J.-H., Wang J., Gao D., Xiong J., Lee P.C. (2022). Al_2_O_3_/HfO_2_ Nanolaminate Dielectric Boosting IGZO-Based Flexible Thin-Film Transistors. Nano-Micro Lett..

[79] Xin S., Chang Y., Zhou R., Cong H., Zheng L., Wang Y., Qin Y., Xu P., Liu X., Wang F. (2023). Ultraviolet-driven metal oxide semiconductor synapses with improved long-term potentiation. J. Mater. Chem. C.

[80] Wang Y., Zhou R., Cong H., Chen G., Ma Y., Xin S., Ge D., Qin Y., Ramakrishna S., Liu X. (2023). Weak UV-Stimulated Synaptic Transistors Based on Precise Tuning of Gallium-Doped Indium Zinc Oxide Nanofibers. Adv. Fiber Mater..

[81] Liu C., Wang Z., Zhang Y., Lü H., Zhang Y.-M. (2022). Nanolaminated HfO_2_/Al_2_O_3_ Dielectrics for High-Performance Silicon Nanomembrane Based Field-Effect Transistors on Biodegradable Substrates. Adv. Mater. Interfaces.

